# Construction of stable mouse artificial chromosome from native mouse chromosome 10 for generation of transchromosomic mice

**DOI:** 10.1038/s41598-021-99535-y

**Published:** 2021-10-08

**Authors:** Satoshi Abe, Kazuhisa Honma, Akane Okada, Kanako Kazuki, Hiroshi Tanaka, Takeshi Endo, Kayoko Morimoto, Takashi Moriwaki, Shusei Hamamichi, Yuji Nakayama, Teruhiko Suzuki, Shoko Takehara, Mitsuo Oshimura, Yasuhiro Kazuki

**Affiliations:** 1 Chromosome Engineering Research Center, Tottori University, 86 Nishi-cho, Yonago, Tottori 683-8503 Japan; 2 Trans Chromosomics, Inc., 86 Nishi-cho, Yonago, Tottori 683-8503 Japan; 3 Division of Genome and Cellular Functions, Department of Molecular and Cellular Biology, School of Life Science, Faculty of Medicine, Tottori University, 86 Nishi-cho, Yonago, Tottori 683-8503 Japan; 4 Division of Radioisotope Science, Research Initiative Center, Organization for Research Initiative and Promotion, Tottori University, 86 Nishi-cho, Yonago, Tottori 683-8503 Japan; 5 Stem Cell Project, Tokyo Metropolitan Institute of Medical Science, 2-1-6 Kamikitazawa, Setagaya-ku, Tokyo, 156-8506 Japan

**Keywords:** Experimental organisms, Gene delivery, Genetic engineering, Genetic techniques, Animal biotechnology, Gene delivery, Genomics, Cytogenetics, Genomics, Chromosomes

## Abstract

Mammalian artificial chromosomes derived from native chromosomes have been applied to biomedical research and development by generating cell sources and transchromosomic (Tc) animals. Human artificial chromosome (HAC) is a precedent chromosomal vector which achieved generation of valuable humanized animal models for fully human antibody production and human pharmacokinetics. While humanized Tc animals created by HAC vector have attained significant contributions, there was a potential issue to be addressed regarding stability in mouse tissues, especially highly proliferating hematopoietic cells. Mouse artificial chromosome (MAC) vectors derived from native mouse chromosome 11 demonstrated improved stability, and they were utilized for humanized Tc mouse production as a standard vector. In mouse, however, stability of MAC vector derived from native mouse chromosome other than mouse chromosome 11 remains to be evaluated. To clarify the potential of mouse centromeres in the additional chromosomes, we constructed a new MAC vector from native mouse chromosome 10 to evaluate the stability in Tc mice. The new MAC vector was transmitted through germline and stably maintained in the mouse tissues without any apparent abnormalities. Through this study, the potential of additional mouse centromere was demonstrated for Tc mouse production, and new MAC is expected to be used for various applications.

## Introduction

Human artificial chromosome (HAC) vectors derived from native human chromosomes, independently and stably maintained without disrupting host chromosomes, have capacities to carry desired copy number of genes via recombination sites, and transfer a large quantity of Mb-sized genes^1^. Taking these advantages over conventional vectors, transchromosomic (Tc) mice into which arbitrary genes were introduced via the HAC vectors, have been generated^2,3^. However, in an attempt to create a mouse model, the HAC vectors have demonstrated a variable retention rate in tissues of mouse individuals, and that stability is particularly low in hematopoietic cells with high proliferation^4^. To address this issue, we established a chromosome transfer- and recombinase-mediated genomic transfer (CT-RMGT) method that maintains an arbitrary human chromosomal region at the chromosome end of the mouse host via chromosome engineering technologies^5^. As a result, although stability was ensured, the method was labor-intensive and time-consuming, which was a barrier to constantly generate the mouse models.

Under these circumstances, a strategy to design a stable mouse artificial chromosome (MAC) vector was conceived through construction of a MAC vector from native mouse chromosome 11 based on the hypothesis that the centromere from own species contributes to high stability (hereinafter referred to as 11MAC)^4^. As in the construction of HAC vectors, the MAC vectors have a structure in which endogenous gene region was deleted by telomere truncation, and the recombination acceptor site capable of gene loading and translocation cloning was inserted. The MAC vectors have the same advantages as the HAC vectors, and additionally have been shown to be uniformly and stably maintained in the mouse tissues including hematopoietic cells^4^. Several Tc mouse models for prediction of human pharmacokinetics and aneuploidy syndrome have been generated by using the 11MAC vectors^6–10^. Furthermore, the 11MAC was found to be stable in rat tissues by generating Tc rat models for human pharmacokinetics^8^.

Importantly, the 11MAC vector is stably maintained in the tissues of Tc mouse as the 41st chromosome. In the Tc mice, two pairs of mouse chromosome 11 with endogenous genes and additional mouse chromosome 11 centromere with no genes are held. In particular, it has been revealed that the 11MAC vector itself does not affect development and homeostasis of the mouse^3^. However, it remains unclear whether MAC vector derived from native mouse chromosome other than mouse chromosome 11 are similarly and stably maintained in the mouse tissues as the 41st chromosome. Further examples are needed to clarify the original hypothesis.

To investigate this, a new MAC with different centromere from other chromosomes excluding native mouse chromosome 11 was required. Current understandings of mouse chromosomes suggest mouse centromere on each chromosome is composed of different copy number of core centromere (minor: 120 bp) satellite and pericentromeric (major: 234 bp) satellite repeat except Y chromosome as different signal intensities or colors were observed on each chromosome in quantitative fluorescence in situ hybridization (Q-FISH) or multicolor FISH (mFISH) images of mouse embryonic fibroblast (MEF) or mouse embryonic stem (mES) cells^4,11–13^. Additionally, the previous analysis of composition of genetic materials on chromosomes suggested that mouse chromosome 10 was more recently reorganized based on evolutionary studies^14,15^. Therefore, considering the potential differences in composition of centromere sequence and chromosome evolution, we selected mouse chromosome 10 to construct new MAC. In addition, construction of MAC vector needed the native mouse chromosome tagged with drug selection marker to transfer the chromosome to other cells for efficient modification with conventional chromosome engineering techniques.

In this study, to elucidate the above, we constructed a new MAC vector from native mouse chromosome 10 (10MAC), and produced Tc mice carrying the 10MAC. The 10MAC was constructed through the whole cell fusion and chromosome transfer with microcell-mediated chromosome transfer (MMCT) to the cells for chromosome modification. Constructed 10MAC1 and other divergent vectors were transferred to CHO cells for testing the gene loading capacity as gene delivery vectors. The 10MAC1 was further transferred to mouse embryonic stem cells to produce the Tc mice. Retention and structure of mouse chromosome 10 and 10MAC vectors in the host cells and mice were visibly analyzed by FISH. We also confirmed that the 10MAC1 can be stably retained in the mouse tissues with GFP monitoring system and does not affect the development and homeostasis of the mouse. Taken together, we here demonstrated the generation of a functional MAC vector underived from mouse chromosome 11, and verified the capability of 10MAC vectors for generation of Tc mice.

## Results

### Generation of a 10MAC

**Figure 1:**
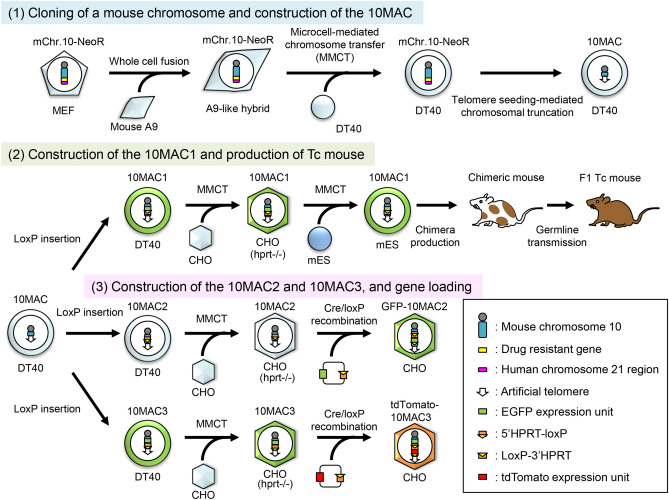
Schematic diagram outlining the construction of new MAC vectors. (1) Cloning of a mouse chromosome and construction of the 10MAC vector. Mouse embryonic fibroblasts (MEFs) containing the NeoR-tagged mouse chromosome 10 (mChr.10-NeoR) were fused with mouse A9 cells. The mChr.10-NeoR was transferred from whole cell hybrids into DT40 cells by microcell-mediated chromosome transfer (MMCT), and the DT40 microcell hybrids containing the mChr.10-NeoR were designated DT40 mChr.10-NeoR. Chromosome manipulation was performed in the homologous recombination-proficient DT40 microcell hybrids. The distal q-arm was deleted from the mChr.10-NeoR by telomere seeding-mediated chromosomal truncation. This mini-chromosome was designated as 10MAC. (2) Construction of the 10MAC1 vector and production of Tc mice. The plasmid containing EGFP and loxP-3’HPRT genes were cloned into a specific site of the 10MAC vector in DT40 cells by homologous recombination. The MAC vector containing EGFP and loxP site was designated as 10MAC1 vector. The EGFP can monitor the existence of 10MAC1 vector. The 10MAC1 was transferred into mouse embryonic stem (ES) cells via CHO cells for subsequent studies. To investigate the stability of the 10MAC1 vector, chimeric mice were produced from the ES cells containing the 10MAC1. The F1 mice were obtained by mating between chimeric and wild-type mice. (3) Construction of the 10MAC2 and 10MAC3 vectors and Cre/loxP-mediated gene loading. The 5’HPRT-loxP plasmid was targeted to a proximal region of the q-arm of the 10MAC vector in DT40 cells (10MAC2). The 10MAC2 vector was transferred into CHO (hprt−/−) cells and the circular vector carrying EGFP and loxP-3’HPRT was loaded into the 10MAC2 vector. The plasmid carrying EGFP and 5’-HPRT loxP was targeted to the MAC vector in DT40 cells (10MAC3). The 10MAC3 vector was transferred into CHO (hprt−/−) cells and the circular vector containing loxP-3’HPRT and tdTomato was loaded into the 10MAC3 vector.

**Figure 2:**
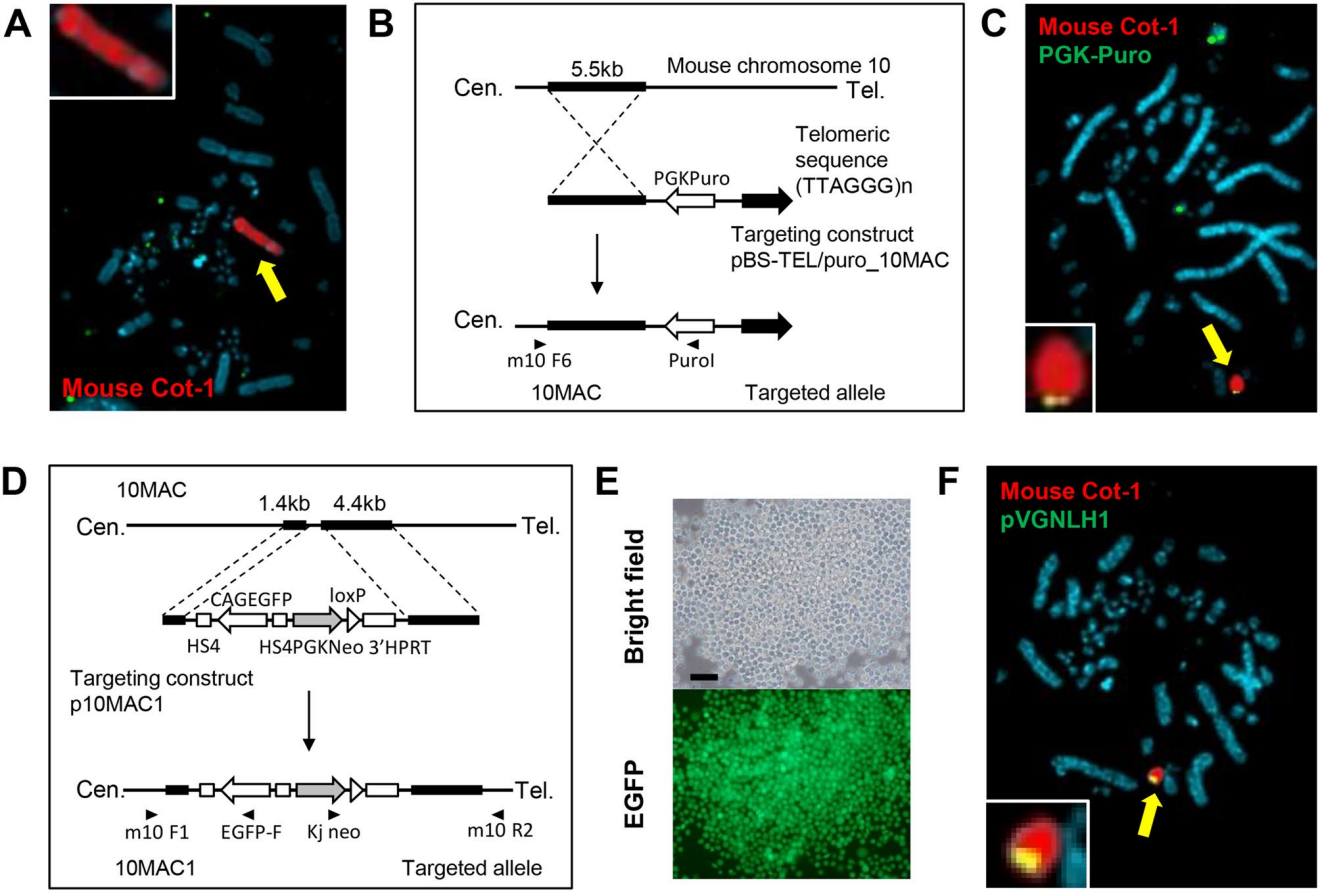
Strategy for the targeted truncation and construction of 10MAC1 vector. **(A)** Fluorescence in situ hybridization (FISH) analysis with a digoxigenin-labeled mouse Cot-1 DNA (red) identified the intact mouse chromosome 10 in the DT40 cells. The arrow indicates an intact mouse chromosome 10 and the inset shows an enlarged image. The chromosomal DNA was counterstained with DAPI, and DAPI was used for the same purpose in the following FISH experiments. **(B)** Strategy for the targeted truncation of the distal region of mouse chromosome 10 by the targeting vector, pBS-TEL/puro_10MAC. The telomere-seeding vector was electroporated into DT40 cells containing mChr.10-NeoR to yield puromycin-resistant transfectants. Arrowheads indicate genomic PCR primers. **(C)** A two-color FISH probe comprising the mouse Cot-1 DNA (red) that hybridizes with the 10MAC and puromycin-resistant gene (green) localized to the distal end of truncated 10MAC. The arrow indicates the MAC fragment, and the inset shows an enlarged image. **(D)** Strategy for the targeted integration of the EGFP gene, NeoR and loxP-3’HPRT into the 10MAC to construct 10MAC1 by the targeting vector, p10MAC1. Arrowheads indicate genomic PCR primers. **(E)** Electroporation of the pMAC1 plasmid yielded GFP-expressing, G418-resistant transfectants from the DT40 (10MAC1) cells. Phase-contrast (top panel) and fluorescence (bottom panel) micrographs are shown. Scale bar: 100 μm. **(F)** FISH analysis of the 10MAC1 vector in the DT40 (10MAC1) cells using the digoxigenin-labeled mouse Cot-1 DNA (red) and the biotin-labeled pVGNLH1 (green). The arrow indicates 10MAC1, and the inset shows an enlarged image of 10MAC1.

We performed the following experiments to generate MAC from native mouse chromosome 10 by chromosome engineering (Fig. 1). Mouse A9 cells are good donors for MMCT owing to the property of high micronuclei formation induced by colcemid treatment. Therefore, MEFs, in which endogenous mouse chromosome 10 (mChr.10) was tagged with NeoR gene (mChr.10-NeoR), were fused with mouse A9 cells carrying Bsd gene. We obtained mouse A9-like whole cell hybrids resistant to both G418 and Blasticidin S. Then, the mChr.10-NeoR was transferred from the hybrid cells to homologous recombination-proficient chicken DT40 cells via MMCT. DT40 cells are useful host cells for efficient chromosome modification. FISH analysis confirmed that the mChr.10-NeoR was independently and stably maintained in DT40 hybrid cells (Fig. 2A).

To truncate endogenous gene-coding region of mChr.10, pericentromeric region was targeted by artificial telomere seeding technique (Fig. 2B). The targeting vector carrying the puromycin resistant gene was introduced into DT40 mChr.10-NeoR by electroporation. As a result of screening drug-resistant clones by PCR and FISH analyses, deletion of a region distal to the target sequence of mChr.10 (hereafter referred as 10MAC) was confirmed in 4 out of 266 clones (DT40 10MAC) (Fig. 2C). In the obtained DT40 10MAC clones, deletion of the endogenous gene-coding region and NeoR gene resulted in an empty artificial chromosome composed of centromere and telomeres.

### Construction of MAC capable of gene loading by recombination system

Consistent with the past artificial vectors we designed, we attempted to construct an artificial chromosome vector capable of carrying desired genes via Cre/loxP recombination system. First, a 10MAC vector carrying CAG-EGFP flanked by insulators, NeoR gene and loxP-3’HPRT was constructed (Fig. 2D). Regarding the gene loading on the 10MAC vectors and selection system, we utilized HPRT gene reconstitution system with HAT (hypoxanthine, aminopterin and thymidine) selection in CHO hprt−/− cells^16,17^. First half unit is PGK promoter-5’UTR-HPRT exon 1–2-splice donor-intron-loxP (5’HPRT-loxP), and another unit is loxP-intron-splice acceptor-HPRT exon 3–9-poly A signal (loxP-3’HPRT). To survive and proliferate, CHO hprt−/− cells required HPRT gene, hypoxanthine and thymidine via HPRT-contributed salvage pathway under the presence of de novo nucleoside biosynthesis pathway inhibitor, aminopterin. If successful gene loading occurred by Cre/loxP system, concatenation of 5’HPRT-loxP and loxP-3’HPRT on either 10MAC or gene loading vector/donor chromosome would result in HPRT expression, which conferred HAT resistance to CHO hprt−/− cells for drug screening. After electroporation of targeting construct to DT40 10MAC, drug selection was performed with G418, and 24 clones, positive for EGFP fluorescence and drug resistance, were randomly selected for further screening (Fig. 2E). Eight out of 24 clones were PCR positive, and as demonstrated by FISH analysis, in all 8 clones, a signal indicating the presence of the loaded unit was confirmed on the MAC maintained independently of the host chromosome (DT40 10MAC1) (Fig. 2F).

**Figure 3:**
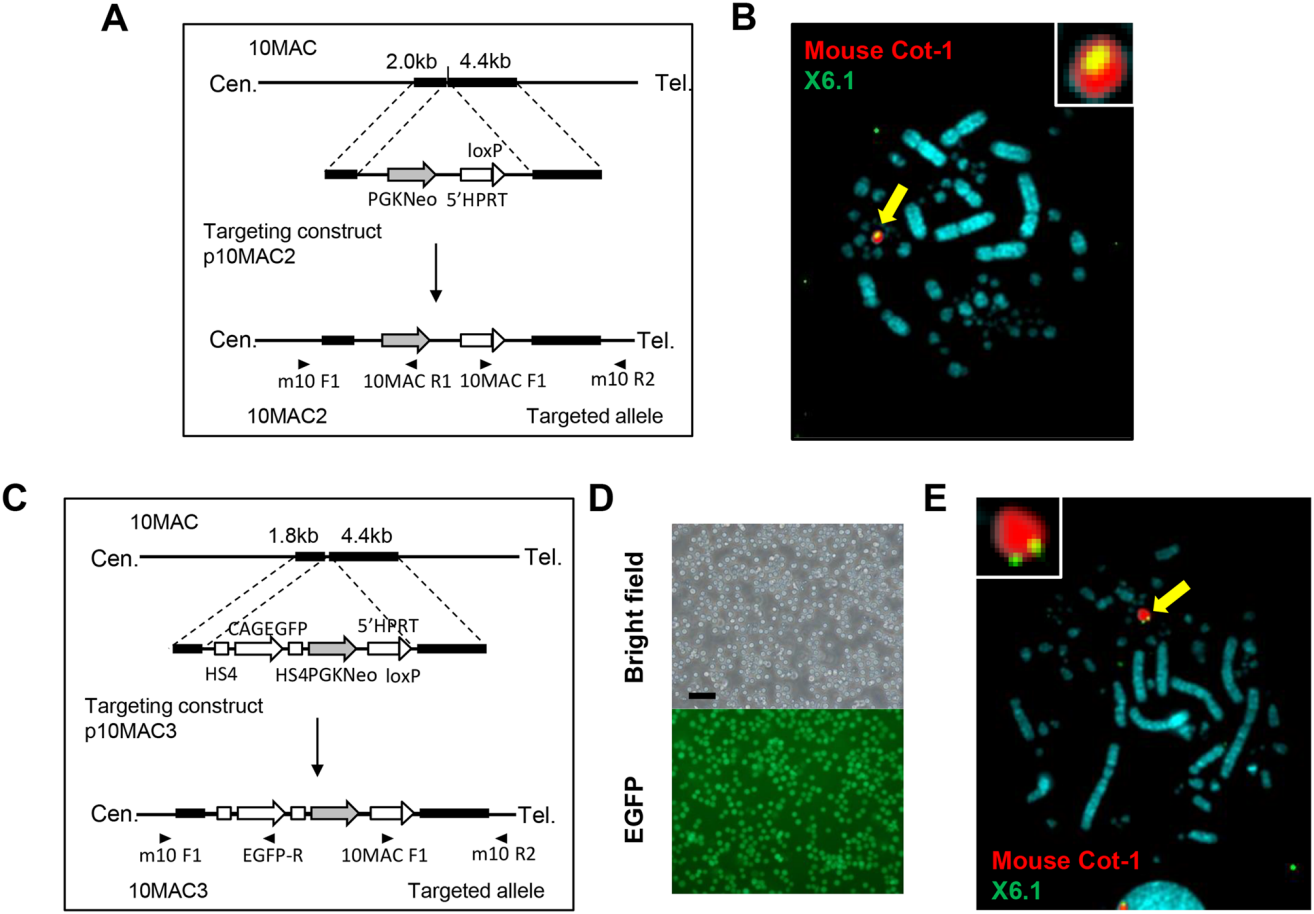
Strategy for the construction of 10MAC2 and 10MAC3 vectors. **(A)** Strategy for the targeted integration of NeoR and 5’HPRT-loxP into the 10MAC to generate 10MAC2 vector by the targeting vector, p10MAC2. Arrowheads indicate genomic PCR primers. **(B)** FISH analysis of the 10MAC2 vector in the DT40 (10MAC2) cells using the digoxigenin-labeled mouse Cot-1 DNA (red) and the biotin-labeled 5’HPRT-loxP gene (green). The arrow indicates the 10MAC2 vector, and the inset shows an enlarged image of the 10MAC2 vector. **(C)** Strategy for the targeted integration of EGFP, NeoR and 5’HPRT-loxP into the 10MAC to generate 10MAC3 vector by the targeting vector, p10MAC3. Arrowheads indicate genomic PCR primers. **(D)** Electroporation of the p10MAC3 plasmid yielded GFP-expressing, G418-resistant transfectants from the DT40 (10MAC3) cells. Phase-contrast (top panel) and fluorescence (bottom panel) micrographs are shown. Scale bar: 100 μm. **(E)** FISH analysis of the 10MAC3 vector in the DT40 (10MAC3) cells using the digoxigenin-labeled mouse Cot-1 DNA (red) and the biotin-labeled 5’HPRT-loxP gene (green). The arrow indicates the 10MAC3 vector, and the inset shows an enlarged image of the 10MAC3 vector.

Next, 10MAC vector carrying NeoR gene and 5’HPRT-loxP was constructed (Fig. 3A). A targeting construct was introduced into DT40 10MAC by electroporation, and 23 clones were randomly selected from the obtained drug resistant clones. Twenty one out of 23 clones were PCR positive. Five clones were selected from PCR positive clones and FISH analysis was performed. We confirmed the presence of the platform inserted on the 10MAC that was maintained independently in all clones (DT40 10MAC2) (Fig. 3B).

Furthermore, the 10MAC which contains CAG-EGFP flanked by insulators, NeoR gene and 5’HPRT-loxP was constructed (Fig. 3C). A targeting construct was introduced into DT40 10MAC, and 24 EGFP fluorescence positive and drug resistant clones were randomly selected (Fig. 3D). PCR analysis was performed, and 23 clones were confirmed to be PCR positive. Among them, 5 clones were selected and analyzed by FISH. FISH analysis revealed that the expected targeting occurred on the 10MAC for all clones (DT40 10MAC3) (Fig. 3E). As a result, three types of 10MAC vectors with different platforms were constructed (Supplementary Fig. S1).

### Transfer of 10MAC1 to CHO hprt−/− cell line and verification of site-specific recombination

**Figure 4:**
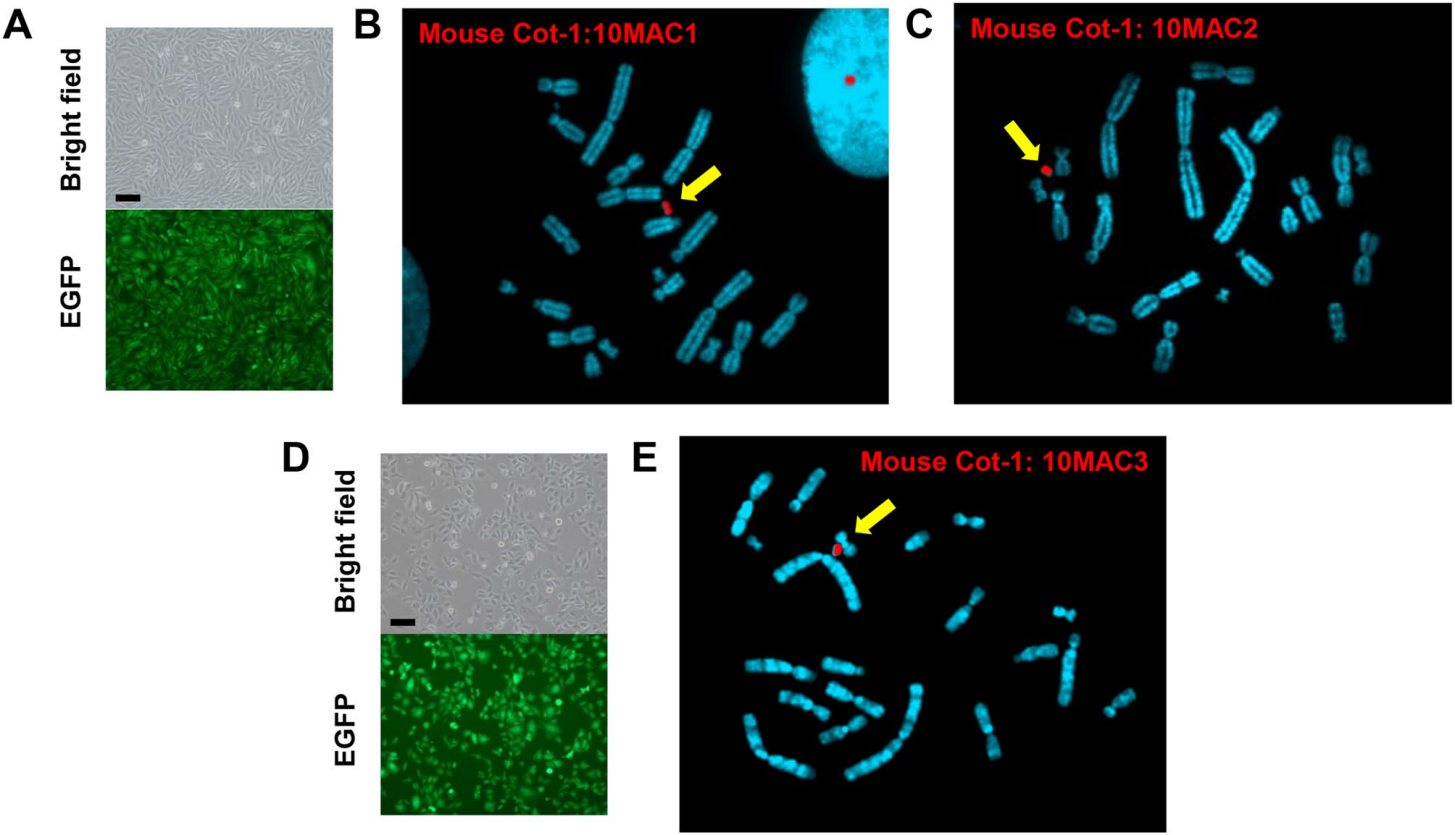
Transfer of 10MAC vectors to CHO (hprt−/−).** (A,D)** Phase-contrast (top panel) and fluorescence (bottom panel) micrographs of CHO (10MAC1) and CHO (10MAC3) are shown. Scale bar: 100 μm. **(B,C,E)** FISH analyses of each 10MAC vector in the CHO (10MAC1), CHO (10MAC2) and CHO (10MAC3) by digoxigenin-labeled mouse Cot-1 DNA. Arrows indicate each 10MAC vector.

The loxP-3’HPRT unit on the 10MAC1 is utilized for the reconstitution of HPRT gene and enables HAT selection when the desired gene with 5’HPRT-loxP unit is loaded via Cre/loxP system. Therefore, 10MAC1 was transferred from DT40 to CHO hprt−/−, which is a Hprt gene-deficient cell line, by MMCT. Six clones of EGFP fluorescence positive and G418 resistant CHO hybrids were obtained (Fig. 4A). Although all clones were positive for PCR analysis, FISH analysis confirmed that one clone retained one copy of 10MAC1 independent of the host chromosome (CHO 10MAC1) (Fig. 4B). To evaluate whether the gene loading system functions on the 10MAC1, a plasmid carrying the 5’HPRT-loxP unit and Cre expression vector were co-transfected into CHO 10MAC1. PCR analysis of 24 randomly selected drug resistant clones revealed that all of them were positive for recombination junction PCR, which indicated that the system worked (Supplementary Table S2). Therefore, it was shown that the constructed 10MAC1 can be monitored by EGFP and gene loading can be performed efficiently.

### Transfer of 10MAC2 and 10MAC3 to CHO hprt−/− cells and verification of gene loading

Following the strategy used to generate 10MAC1, 10MAC2 and 10MAC3 were transferred from DT40 cells to CHO hprt−/− cells by MMCT. Regarding the 10MAC2, 11 out of 12 drug-resistant CHO hybrids were PCR positive. FISH analysis was performed on 6 randomly selected clones, and it was confirmed that 3 clones maintained one copy of the 10MAC2 independent from the host chromosome (Fig. 4C). For the 10MAC3, 9 out of 10 clones of EGFP fluorescence positive and drug resistant CHO hybrids were PCR positive (Fig. 4D). FISH analysis revealed that 2 out of 6 clones were desired clones carrying a single copy of the 10MAC3 independent of the host (Fig. 4E). To check the stability of 10MAC vectors in the host cells from different species, Chinese hamster, the long-term culture under the presence or absence of selection drug was performed. The results obtained by FISH and FCM analyses showed that each MAC was relatively stable even under the absence of selection drug, and retention was almost 100% in the presence of selection drug (Supplementary Fig. S2).

**Figure 5:**
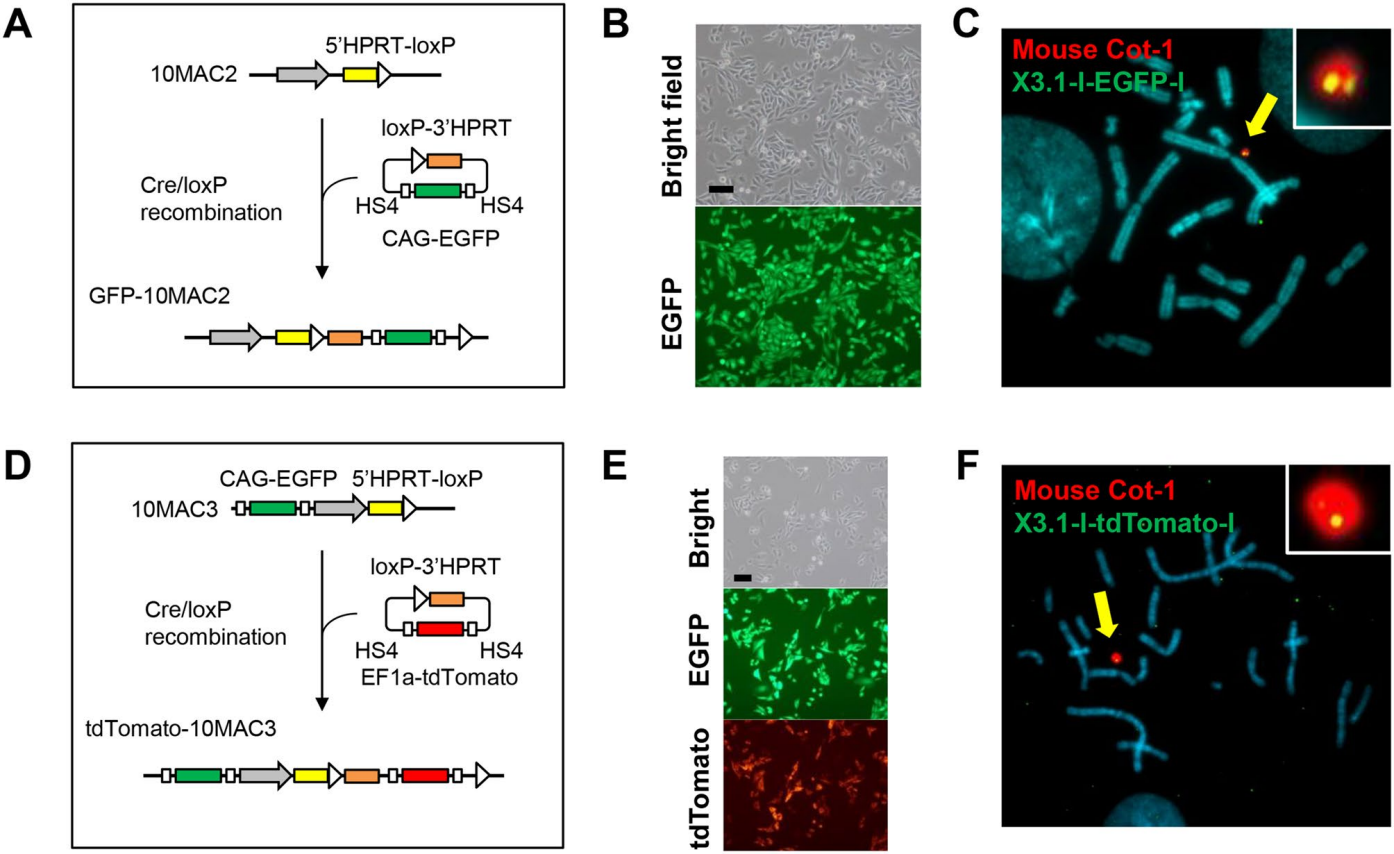
Cre/loxP-mediated gene loading to the 10MAC2 and 10MAC3. **(A,D)** Strategy for the site-specific insertion of the EGFP and tdTomato genes into the 10MAC2 and 10MAC3 vectors, respectively, using the Cre/loxP-mediated system in CHO cells. **(B)** Transfection yielded GFP-expressing HAT-resistant transfectants from the CHO (10MAC2) cells. Phase-contrast (top panel) and fluorescence (bottom panel) micrographs are shown. Scale bar: 100 μm. **(C)** FISH analysis of the GFP-10MAC2 in the CHO (GFP-10MAC2) cells using the digoxigenin-labeled mouse Cot-1 DNA (red) and the biotin-labeled EGFP gene (green). The arrow indicates the GFP-10MAC2 vector, and the inset shows an enlarged image of the GFP-10MAC2 vector. **(E)** Transfection yielded tdTomato-expressing HAT-resistant transfectants from the CHO (10MAC3) cells. Phase-contrast (top panel) EGFP (middle panel) and tdTomato (bottom panel) micrographs are shown. Scale bar: 100 μm. **(F)** FISH analysis of the tdTomato-10MAC3 in the CHO (tdTomato-10MAC3) cells using the digoxigenin-labeled mouse Cot-1 DNA (red) and the biotin-labeled tdTomato gene (green). The arrow indicates the tdTomato-10MAC3 vector, and the inset shows an enlarged image of the tdTomato-10MAC3 vector.

For both 10MAC2 and 10MAC3, we verified whether gene-loaded clone could be obtained by HPRT reconstruction using the Cre/loxP system. A vector carrying loxP-3’HPRT and EGFP expression units and a vector containing loxP-3’HPRT and tdTomato expression units were transfected into CHO 10MAC2 and CHO 10MAC3, respectively, with the Cre expression vector (Fig. 5A,D and Supplementary Table S2). We obtained HAT-resistant clones positive for EGFP and tdTomato fluorescence (Fig. 5B,E). Furthermore, FISH analysis was performed to confirm the existence of on-board gene units on respective 10MAC vectors, excluding the possibility of random integration to host chromosome (Fig. 5C,F). Since signal of the loaded genes was detected on respective 10MACs, these results suggested that the gene-loading system worked as expected. Taken together, both 10MAC2 and 10MAC3 are capable of gene-loading using the Cre/loxP system.

### Transfer of the 10MAC1 vector to mouse embryonic stem cells and Tc mice generation

**Figure 6:**
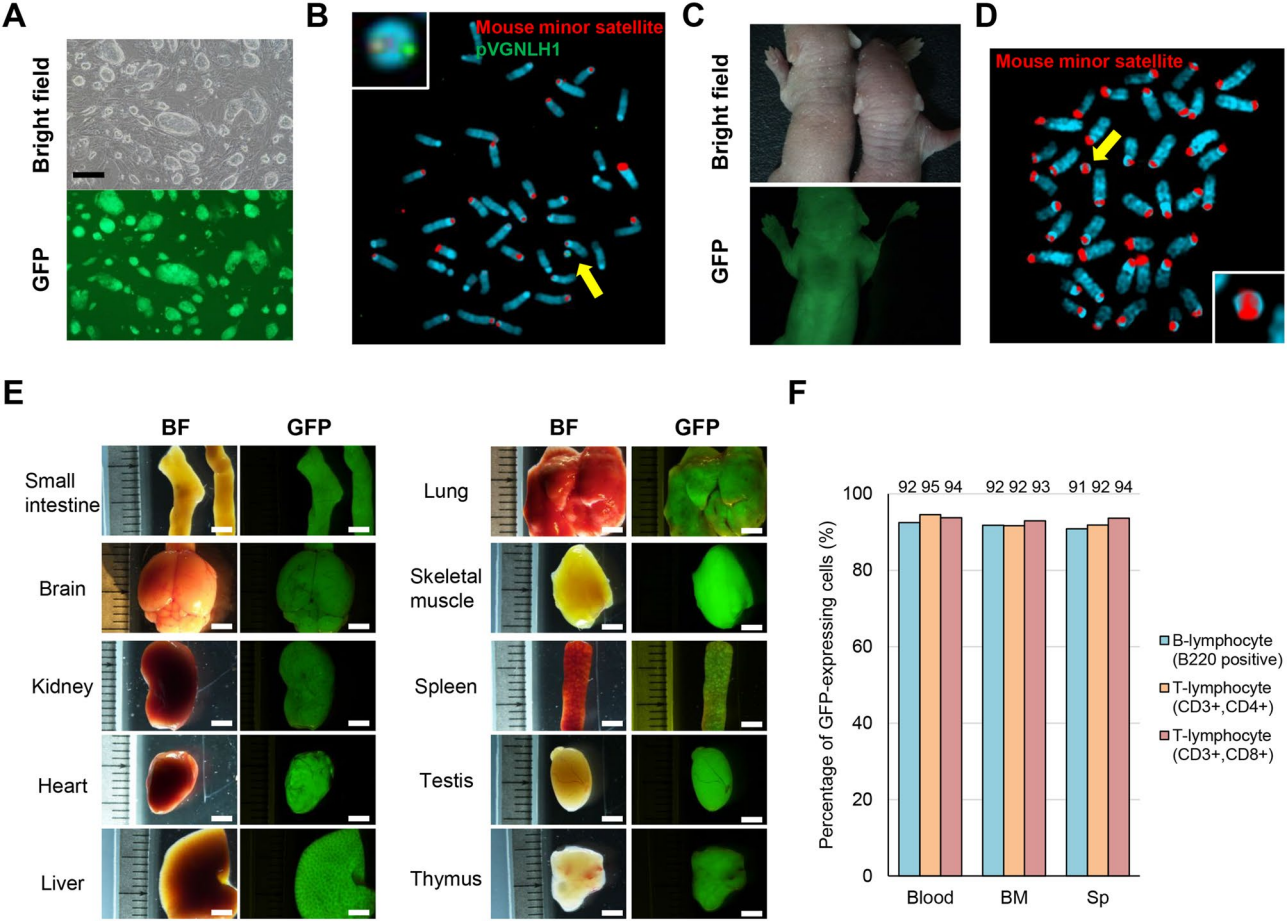
Generation of Tc mice carrying 10MAC1 and its stability in the tissues. **(A)** Phase-contrast (top panel) and fluorescence (bottom panel) micrographs of mouse ES hybrids carrying 10MAC1 are shown. Scale Bar: 100 μm. **(B)** FISH analysis of the mouse ES hybrids carrying 10MAC1 using the digoxigenin-labeled mouse minor satellite DNA (red) and the biotin-labeled pVGNLH1 (green). Inset shows an enlarged image of the 10MAC1. **(C)** Phase-contrast (top panel) and fluorescence (bottom panel) micrographs of wild-type (right) and Tc mice (left) are shown. **(D)** Representative FISH image of metaphase chromosome spreads of lymphocytes in blood from Tc mice using the digoxigenin-labeled mouse minor satellite DNA (red). Inset shows an enlarged image of the 10MAC1. **(E)** GFP images of different tissues from Tc mice carrying the 10MAC1. GFP expression indicates the presence of 10MAC1. Exposure times for each GFP image of small intestine, brain, kidney, heart, liver, lung, skeletal muscle, spleen, testis, and thymus were 100, 500, 200, 100, 400, 700, 100, 400, 200 and 200 ms, respectively. Scale bar: 3 mm. **(F)** Percentage of GFP-expressing lymphocyte subsets from blood, bone marrow and spleen.

To evaluate the stability of the 10MAC1 derived from native mouse chromosome 10 in mice, we attempted to generate Tc mouse carrying the 10MAC1. Chromosome transfer was performed by standard PEG-MMCT and retro-MMCT from CHO cells to mouse ES cells (39,XO) and drug-resistant and GFP-positive clones were selected for further analyses (Fig. 6A). Retro-MMCT is a highly efficient chromosome transfer method reported recently, which can reduce donor scale^18^. FISH analysis with the digoxigenin-labeled mouse minor satellite and biotin-labeled platform construct (pVGNLH1) probes showed that a single copy of 10MAC1 was independently maintained in mES hybrids (Fig. 6B). Among them, we selected clones with desired karyotype (40,XO, + 10MAC1) (TT2F 10MAC1). Then, we produced female chimeric mice with high coat-color chimerism by injecting TT2F 10MAC1 into eight-cell-stage embryo. These chimeric mice were crossed with wild-type mice to verify whether the 10MAC1 derived from native mouse chromosome 10 is transmitted through germline. Among the offsprings, GFP-positive mice with the 10MAC1 retention were obtained without any apparent abnormality (Fig. 6C). To confirm the independent maintenance of 10MAC1 from host chromosome, the metaphase spreads of lymphocytes in blood collected from Tc mice were analyzed. The 10MAC1 was single copy and independently maintained in host cells without integration (Fig. 6D). These results suggested that the 10MAC1 can be transmitted through germline and independently maintained without affecting the development of Tc mouse.

### Retention rate of the 10MAC1 vector in mouse

To investigate whether 10MAC1 derived from native mouse chromosome 10 is stable in the mouse tissues in the identical manner as previously constructed 11MAC derived from native mouse chromosome 11, we analyzed Tc mice carrying 10MAC1 vector. The EGFP on the 10MAC1 was expressed in all tissues examined, which is comparable to 11MAC Tc mice (Fig. 6E).

Given the high rate of cellular turnover, hematopoietic cells from blood, bone marrow and spleen are optimal target cells to evaluate the stability of mammalian artificial chromosomes (ACs) in mouse. In previous report, HAC vector was relatively stable in various tissues except hematopoietic cells in Tc mice. In contrast, 11MAC derived from native mouse chromosome 11 is highly stable in all tissues examined including hematopoietic cells in Tc mice^4^. The degree of stability is thought to be more pronounced in the hematopoietic cells with rapid turnover. According to the data from several Tc mouse models generated by HAC and MAC vectors, the retention rate of mammalian ACs in the hematopoietic cells reflects those of mammalian ACs in the whole body.

Considering the reports mentioned above, in this study, we evaluated the stability of 10MAC1 in Tc mice by monitoring GFP-positive cells from blood, bone marrow and spleen by FCM analysis. The stability of 10MAC1 can be evaluated by monitoring GFP-positive cells because the 10MAC1 contains the GFP expression unit driven by constitutive active CAG promoter in common with the 11MAC1 as reported previously^4^. The retention rate of the 10MAC1 was high and uniform in B cells, CD4 and CD8 T cells from peripheral blood, bone marrow and spleen (89–94%) (Fig. 6F). The rates of GFP-positive cells with the 10MAC1 in lymphocytes from bone marrow and spleen were clearly higher than those with HAC (< 20%) and comparable to those with 11MAC1 (> 90%). The mice in minor population that showed lower retention rate of the 10MAC1 in the peripheral blood reflected the lower retention rates among different tissues (Supplementary Fig. S3 and S4). These results strongly support the high stability of the 10MAC1 and significant correlation of 10MAC1 retention rates between peripheral blood and different tissues.

The germline transmission efficiency and stability of 10MAC1 in lymphocytes was evaluated through generations. The germline transmission rates, i.e., 10MAC1-positive rates in offspring from male and female were 11.7% and 32.1–69.2%, respectively. As previously reported, the transmission rates of 11MAC1 to offspring were 30% from male and 50% from female^19^. This non-Mendelian segregation may be due to biological issues associated with fertilization or development, but not meiosis I and II stages. To maintain the strain, we utilized the mice with high GFP-positive rate in blood cells for crossing based on our empirical evidence that mice with high GFP-positive rate tend to give offspring with high GFP-positive rate. Regarding the stability, although some Tc mice showed lower retention rate, main population maintained high stability through generations (Supplementary Fig. S5). These results suggested that the 10MAC1 derived from native mouse chromosome 10 is highly stable in mice.

## Discussion

In this study, we constructed a new MAC vector from native mouse chromosome 10. The 10MAC1 was transmitted through germline in mice and there was no apparent abnormality in Tc mice carrying 10MAC1 through generations. The assessment of stability by FCM analysis of lymphocyte subsets from each tissue clarified the high stability of 10MAC1 even in the hematopoietic cells with high proliferation. From the previous reports, because of the correlation between high proliferation and increased loss of mammalian ACs, the stability of ACs in lymphocyte subset is well established determinant factor reflecting their stability among various tissues in the host individuals^4^. Therefore, 10MAC1 is expected to be available for generating Tc mouse models. Considering the stability in mice, both 10MAC and 11MAC vectors are functionally comparable. The study also indicated that the MAC derived from native mouse chromosome other than mouse chromosome 11 is also highly stable in mice.

Further study is necessary to evaluate the different degrees of stability levels among native mouse centromeres by generating various MACs from other native chromosomes. To this end, multiple technological approaches through the combinations of the recent advancements in genome editing technology and chromosome transfer are expected to promote the research regarding the stability of centromeres in own species. In this analysis, as a starting point to clarify the potential of native mouse centromere for Tc mice production, the MAC vectors constructed herein can be utilized for various purposes including: (1) efficient gene loading system considering compatible constructs prepared previously, (2) cell resource production by transfer of new MACs with functional gene units, and (3) investigation on the compatibility of centromere function beyond species.

The 10MAC vectors described herein offer the following notable features with distinct aims. The 10MAC1 with loxP-3’HPRT is aimed at translocation cloning of desired chromosome fragments with 5’HPRT-loxP such as human native chromosome region to generate a humanized mouse model with gene cluster of Mb-sized gene^16^. The 10MAC2 is for loading general single vector or previously established simultaneous or sequential integration of multiple gene loading vectors (SIM system) which enable us to load multiple vectors sequentially or three vectors simultaneously each round^16,20^. The other gene loading system such as multi-integrase (MI) system also can be applied to 10MAC2 by inserting MI platform with four integrase and one recombinase acceptor sites^21–23^. The 10MAC3 is a chromosomal vector in which an EGFP monitor system is added to 10MAC2.

The 10MAC2 and 10MAC3 are expected to be utilized for not only generating mouse models but also establishing useful cell lines for material production and functional analysis comparable to those described by using 11MAC^4^. Several in vitro models have already been established; i.e., hepatoblastoma cell line, HepG2 carrying the 11MAC with the drug-metabolizing cytochrome P450 (CYP) enzymes for assessing drug metabolism and hepatotoxicity^24–26^, human colon adenocarcinoma cell line, Caco2 carrying the MAC with CYPs for prediction of the absorption and metabolism in the human intestine^27^, luminescent HepG2 with the MAC carrying luciferase reporter units for cytotoxicity assay^26^, immortalized mouse S3 cells carrying the 11MAC with Kidney injury molecule-1 (Kim-1) and Hprt reporter units for assessing nephrotoxicity^28^, and human mesenchymal stem cells (MSCs) carrying the MAC with osteocalcin (OC) reporter for monitoring osteogenic differentiation^29^. Additionally, recent work reported a double 11MAC-retaining cell based model wherein HepG2 cells with multiple CYPs and POR genes on one 11MAC and luminescence expression unit on another were generated for easy real-time monitoring system of hepatotoxicity by molecules^30^. Evidence of their applications with multiple copies also suggested the flexibility of MAC vector system.

The stability of 10MAC vectors in cells and tissues of other species remains to be evaluated. As with 11MAC vectors, 10MAC vectors are presumably stable in human cell lines such as fibrosarcoma HT1080 and hepatoblastoma HepG2^19,25^. In addition, high stability of the 11MAC vectors in the humanized Tc rat models for pharmacokinetics implies the potential of 10MAC vectors for generation of Tc rat models^8^. Establishment of 10MAC-containing cell lines derived from several species may promote investigation on the compatibility between mouse centromere and centromere-associated proteins of host species to search the key factors for chromosome stability. Interestingly, on stability of human chromosomes in the mouse cell line, while human chromosomes 2 and 11 were unstable in the mouse ES cells, HAC derived from human chromosome 14 (SC20) was stable when compared with those chromosomes leading to the concept of the link between the stability and composition of centromeres even in interspecies comparability^31^. Since the study cannot exclude the effect of endogenous genes of intact or partially fragmented chromosomes on stability, constructions of mammalian ACs without endogenous genes may also promote such analyses.

In conclusion, although universal mammalian AC vector, which is highly stable among the species, is still desired and certain chromosomal vectors such as 11MAC is known to be available for mouse and rat, this study further provided the evidence supporting the original conception that construction of artificial chromosomes from own species is also reasonable strategy for generation of Tc animals from desired species. Collectively, the 10MAC vectors constructed in this study is expected to contribute to generation of Tc mouse models and valuable cell resources for biomedical research and development.

## Materials and methods

### Ethics statement

This study was approved by the Institutional Animal Care and Use Committee of Tottori University and the Recombinant DNA Experiment safety Committee of Tottori University (for performing recombinant DNA experiments). All experiments were carried out in compliance with the ARRIVE guidelines. All methods were carried out in accordance with relevant guidelines and regulations.

### Cell culture

The mouse embryonic fibroblasts, mouse A9 cells, and whole cell hybrids were grown in Dulbecco’s modified Eagle’s medium (DMEM; Wako, Tokyo, Japan) supplemented with 10% fetal bovine serum (FBS; Sigma-Aldrich, St. Louis, MO, USA). The DT40 hybrid cells were maintained in Roswell Park Memorial Institute (RPMI) medium 1640 (Wako, Tokyo, Japan) containing 10% FBS, 1% chicken serum (Gibco, ThermoFischer, Waltham, MA, USA), 50 μmol/L 2-mercaptoethanol, and the appropriate antibiotics. The DT40 hybrids containing a single copy of mouse chromosome 10 were produced by MMCT from mouse A9 hybrid cells containing NeoR-tagged mouse chromosome 10 and maintained with 1500 μg/mL G418 (Promega, Madison, WI, USA). The hypoxanthine phosphoribosyltransferase (Hprt)-deficient CHO (JCRB0218) hybrids containing a 10MAC1, 10MAC2 or 10MAC3 were maintained in Ham’s F-12 nutrient mixture (Wako, Tokyo, Japan) containing 10% FBS and 800 μg/mL G418. The parental mouse ES cell line, TT2F, and the microcell hybrid clone, TT2F (10MAC1), were maintained on mitomycin C (Sigma-Aldrich, St. Louis, MO, USA)-treated neomycin-resistant MEFs (Oriental Yeast, Tokyo, Japan) as feeder layers in DMEM with 18% FBS, 1 mM sodium pyruvate (Invitrogen, Carlsbad, CA, USA), 0.1 mM nonessential amino acids (Invitrogen, Carlsbad, CA, USA), 0.1 mM 2-mercaptoehtanol (Sigma-Aldrich, St. Louis, MO, USA), 2 mM l-glutamine (Invitrogen, Carlsbad, CA, USA), and 1,000 units/mL leukemia inhibitory factor (Funakoshi, Tokyo, Japan).

### Construction of targeting vectors

The homologous regions on mouse chromosome 10 are described in Fig. 1. For constructing a telomere truncation vector, pBS-TEL/puro_10MAC, annealed sense and antisense EcoRI/AscI/EcoRI oligos were inserted into EcoRI site of pBS-TEL/Puro vector^32^. Then, a fragment (5.6 kb) of the mouse chromosome 10 region was amplified by PCR with AscI_m10T F2/BamHI_m10T R3 primers, digested with AscI/BamHI and sub-cloned into the AscI/BamHI sites of the pBS-TEL/Puro vector (pBS-TEL/puro_10MAC). The targeting vector, p10MAC1, for introducing EGFP/neo/loxP-3’HPRT was constructed as follows: Two 1.4 kb and 4.4 kb fragments for homologous arms corresponding to the mouse chromosome 10 pericentromeric region were amplified by PCR using the KpnI_m10 LA F/XhoI_m10 LA R primers (2.0 kb) and SalI_m10 RA F/ NotI_m10 RA R primers (4.4 kb), digested with KpnI/XhoI and SalI/NotI, and subcloned into pKO Scrambler V913 backbone vector (Lexicon Genetics, Woodlands, TX, USA). Then, the EGFP/neo/loxP-3’HPRT cassette from pVGNLH1^4^, digested with SalI/AscI, was introduced into XhoI/AscI sites of the vector (p10MAC1). The targeting vector, p10MAC2, for introducing neo/5’HPRT-loxP was constructed as follows: PGKneo and 5’HPRT-loxP fragments were introduced into EcoRI site and AscI/ClaI sites of pKO Scrambler V907 backbone vector (Lexicon Genetics, Woodlands, TX, USA). Then, a 4.4 kb right arm was amplified by PCR using ClaI_m10 RA F/R primers and inserted into ClaI site of the vector (pN5’HLR). A 2.0 kb left arm was amplified by NotI_m10 LA F/SalI_m10 LA R primers and inserted into NotI/SalI sites of pN5’HLR (p10MAC2). The targeting vector, p10MAC3, for introducing EGFP/neo/5’HPRT-loxP was constructed as follows: CAG-EGFP flanked by insulator HS4 was inserted to NotI/SalI sites of pN5’HLR (pN5’HLER). Then, left arm was amplified by NotI_m10 LA F/SalI_m10 LA R primers and a 1.9 kb NotI/PspOMI-digested fragment was introduced to NotI site of the pN5’HLER (p10MAC3). Primer information is available in Supplementary Table S1.

### Transfection of DT40 and CHO cells

The DT40 (mChr.10-neo) hybrid cells were transfected by electroporation of 1 × 10^7^ cells with each NotI-linearized plasmid at 25 μF and 550 V in a 4 mm cuvette using a Gene Pulser (Bio-Rad, Hercules, CA). The cells were resuspended in basic growth medium and aliquoted into 96-well flat-bottomed microtiter plates with serial dilution (Becton–Dickinson, Franklin Lakes, NJ). Next day, the cells were resuspended in selective medium with 0.5 μg/ml puromycin or 1.5 mg/ml G418. Approximately fourteen days later, drug-resistant colonies were picked up and expanded for the following analyses. The CHO cells (2 × 10^6^ cells) containing 10MAC1, 10MAC2 or 10MAC3 were transfected with pBS185 (CMV-Cre) and plasmids containing 5’HPRT-loxP (X6.1), loxP-3’HPRT-I-CAG-EGFP-I (X3.1-I-EGFP-I) or loxP-3’HPRT-I-EF1a-tdTomato-I (X3.1-I-tdTomato-I) by using 20 μL of Lipofectamine 2000 reagent (Invitrogen, Carlsbad, CA, USA) according to the manufacture’s instruction. After 24 h, cells were scaled up, and after 48 h, cultured in the medium containing 1 × HAT (Sigma-Aldrich, St. Louis, MO, USA) for selection. Fourteen days later, drug-resistant colonies were picked up and expanded for further analyses as described below.

### MMCT

The 10MAC1, 10MAC2 and 10MAC3 vectors were transferred from the DT40 cells into the CHO hprt−/− cells using MMCT technology^2^. Briefly, microcells were prepared by centrifugation of DT40 cells attached to flasks (Nalge Nunc, Rochester, NY, USA) coated with poly-l-lysine followed by fusion with 1 × 10^6^ CHO cells using 42% polyethylene glycol 1000 (Wako, Tokyo, Japan). CHO hybrids containing each 10MAC vector were selected with the media containing 800 μg/mL G418 and selected for expansion. CHO cells containing 10MAC1 without and with ecotropic EnvΔR expression were used as donor microcell hybrids in the PEG-MMCT and retro-MMCT as described previously^18,33^. mES XO ES9 hybrids containing 10MAC1 were selected with the media containing 150 μg/mL G418 and picked for expansion.

### Genomic PCR analysis

Genomic DNA was extracted from cell lines using a genomic extraction kit (Gentra Systems, Minneapolis, MN, USA), and PCR was performed as follows. The screening primers for telomere truncation were m10 F6/PuroI, Gm8155 F/R, Iyd F/R and Plekhg1 F/R. The targeting with p10MAC1 was confirmed by using KpnI_m10 LA F/XhoI m10 LA R, m10 F1/EGFP-F and kj neo/m10 R2. The primers for detection of exact targeting with p10MAC2 were m10 F1/10MAC R1 and 10MAC F1/m10 R2. The targeted allele by p10MAC3 was analyzed by using m10 F1/EGFP-R and 10MAC F1/m10 R2. Contamination of DT40 cells after MMCT from DT40 cells to CHO cells were checked by using CENPH Fw/Rv. Contamination of CHO cells after MMCT from CHO cells to mES cells were checked by using Furin F/R. The recombination with Cre/loxP system on each 10MAC vector was detected by TRNS L1/R1. Primer sequences are described in Supplementary Table S1.

### Fluorescence in situ hybridization (FISH) analysis

Preparation of metaphase chromosomes from exponentially growing cell culture was performed according to standard methods. FISH analyses were performed using the digoxigenin-labeled (Roche, Basel, Switzerland) mouse COT-1 DNA (Invitrogen, Carlsbad, CA, USA) or mouse minor satellite DNA, the biotin-labeled PGKpuro, pVGNLH1, X6.1, X3.1-I-EGFP-I, or X3.1-I-tdTomato-I plasmid DNA essentially as described previously^33^. Chromosomal DNA was counterstained with DAPI (Sigma-Aldrich, St. Louis, MO, US). Images were captured using an AxioImagerZ2 fluorescence microscope (Carl Zeiss, Oberkochen, Germany).

### Generation of chimeric mice

Chimeric mice were produced from TT2F hybrids containing 10MAC1. Chimera production was performed as described previously^33^. mES cells were injected into 8-cell stage embryos derived from ICR mice (CLEA, Tokyo, Japan) and then transferred into pseudopregnant ICR females. Almost 100% coat-color chimeric mice were mated with ICR males to obtain Tc mice.

### GFP image capture and FCM analysis of tissues and blood cells

Samples were collected from euthanized Tc mice containing 10MAC1 and their 10MAC1-negative counterpart with isoflurane after perfusion. Bright and GFP images of each tissue were obtained by M205 FA fluorescence stereo microscope (Leica Microsystems, Wetzlar, Germany) with NIS-Elements software (Nikon, Tokyo, Japan). The dissociated cells were treated with ammonium chloride solution for hemolysis and stained with anti-mouse B220/CD45R (Biolegend, San Diego, CA, USA), CD3 (Biolegend, San Diego, CA, USA), CD4 (Biolegend, San Diego, CA, USA) and CD8a (BD Biosciences, San Jose, CA, USA) antibodies conjugated with BV650, BV650, PE and PE-CF594, respectively. All staining samples (1 × 10^6^ cells) were incubated at 4 °C for 30 min in 100 μl FCM buffer (HBSS with 5% FBS and 1 mM EDTA) (Gibco, ThermoFischer, Waltham, MA, USA). DAPI was added to the final suspension to exclude dead cells. Analyses were conducted using a CytoFLEX S (Beckman Coulter, Brea, CA, USA). Percentage of GFP-expressing cells in lymphocytes through generations was analyzed by Gallios (Beckman Coulter, Brea, CA, USA).

## Supplementary Information

Supplementary Information.

## Data Availability

The datasets generated during and/or analyzed during the current study are available from the corresponding author on reasonable request.
