# Supplementary material for: Construction of stable mouse artificial chromosome from native mouse chromosome 10 for generation of transchromosomic mice
Source: Sci Rep. 2021 Oct 8;11:20050. doi: 10.1038/s41598-021-99535-y (PMC8501010; doi:10.1038/s41598-021-99535-y)
Supplement: Supplementary file 1 — Supplementary Information. [file 41598_2021_99535_MOESM1_ESM.pdf]

Construction of stable mouse artificial chromosome from native mouse chromosome 10 for generation of transchromosomic mice

Satoshi Abe[1,2], Kazuhisa Honma[2], Akane Okada[1], Kanako Kazuki[1], Hiroshi Tanaka[2], Takeshi Endo[2], Kayoko Morimoto[2], Takashi Moriwaki[1,3], Shusei Hamamichi[1], Yuji Nakayama[4], Teruhiko Suzuki[5], Shoko Takehara[2], Mitsuo Oshimura[1,2] and Yasuhiro Kazuki[1,3*]

[1]Chromosome Engineering Research Center, Tottori University, 86 Nishi-cho, Yonago, Tottori 683-8503, Japan

[2]Trans Chromosomics, Inc., 86 Nishi-cho, Yonago, Tottori 683-8503, Japan

[3]Division of Genome and Cellular Functions, Department of Molecular and Cellular Biology, School of Life Science, Faculty of Medicine, Tottori University, 86 Nishi-cho, Yonago, Tottori 683-8503, Japan

[4]Division of Radioisotope Science, Research Initiative Center, Organization for Research Initiative and Promotion, Tottori University, 86 Nishi-cho, Yonago, Tottori 683-8503, Japan

[5]Stem Cell Project, Tokyo Metropolitan Institute of Medical Science, 2-1-6 Kamikitazawa, Setagaya-ku, Tokyo 156-8506, Japan

***Corresponding author:**

Yasuhiro Kazuki, Ph.D.
Division of Genome and Cellular Functions,
Department of Molecular and Cellular Biology,
School of Life Science, Faculty of Medicine,
Tottori University, 86 Nishi-cho, Yonago, Tottori 683-8503, Japan
Phone: +81-859-38-6219, Fax: +81-859-38-6210
E-mail: kazuki@tottori-u.ac.jp

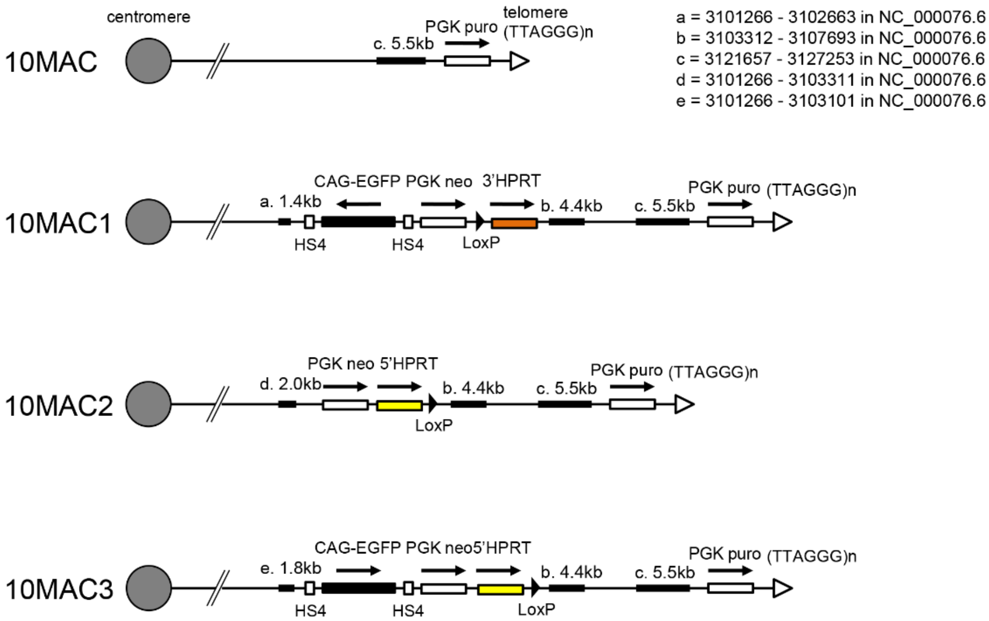

Supplementary Figure S1. Detailed maps of the MACs

(a-e) Homologous region on mChr.10 for gene targeting. EGFP-NeoR-loxP-3'HPRT, NeoR-5'HPRT-loxP and EGFP-NeoR-5'HPRT-loxP were inserted to generate 10MAC1, 10MAC2 and 10MAC3, respectively. The targeting vectors are described in Figs. 2 and 3.

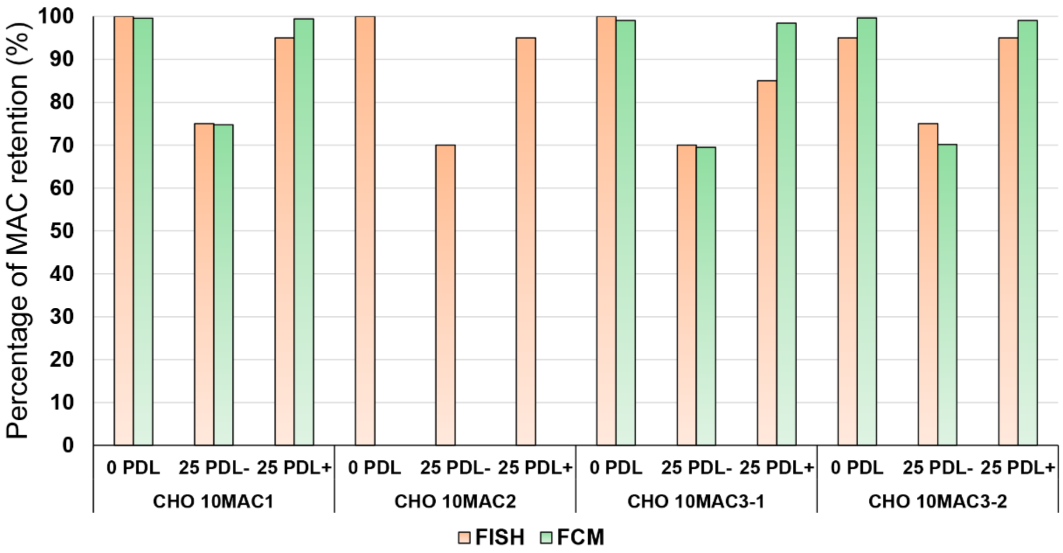

Supplementary Figure S2. Stability of each MAC in CHO hprt-/- cells

Retention rate of each MAC was analyzed by FISH and FCM after long-term culture under the presence or absence of drug selection (minus or plus). FISH analyses determined retention rate of each MAC viewing metaphase spreads and FCM analyses determined retention rate of each MAC by monitoring GFP-expressing cells. Data from FISH and FCM demonstrated high correlations.

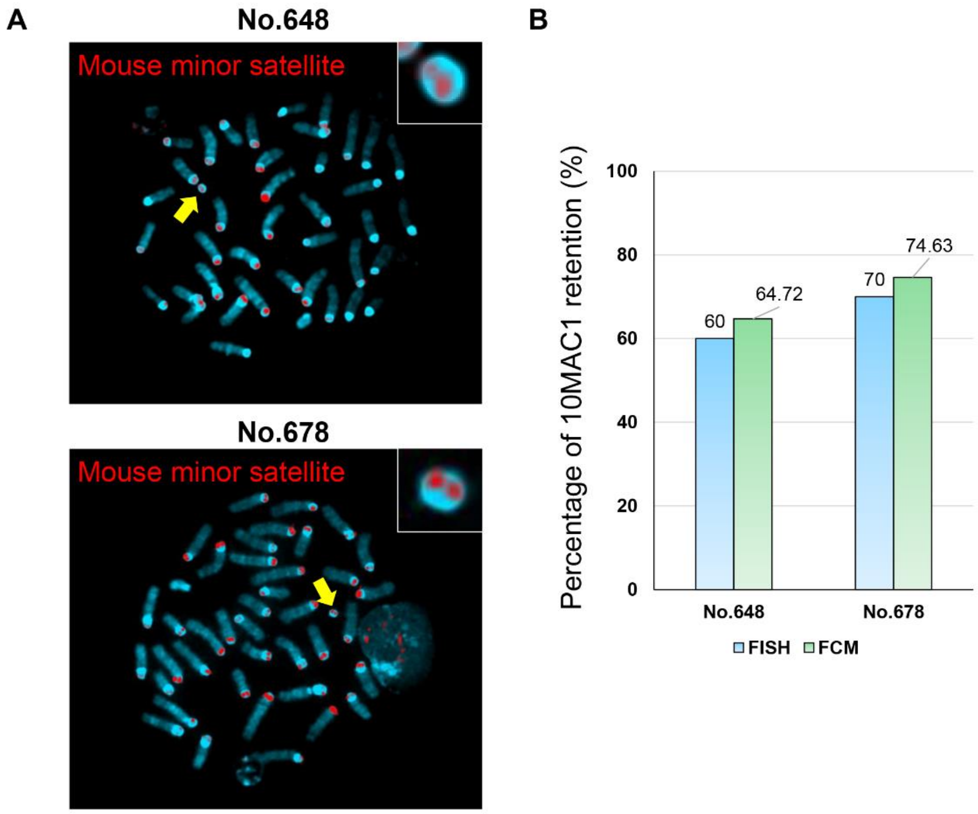

Supplementary Figure S3. Retention rate of the 10MAC1 in minor population of animals with low percentages of GFP-expressing cells.

(A) Representative FISH images of metaphase spreads of cultured lymphocytes from Tc mice with low percentages of GFP-expressing blood lymphocytes (64.72% and 74.63%, respectively). The arrow indicates the 10MAC1 vector, and the inset shows an enlarged image of the 10MAC1 vector. (B) Retention rate of the 10MAC1 as determined by FISH and FCM analyses. Thirty metaphases were analyzed for each Tc mouse. Collectively, FISH and FCM data demonstrated high correlations.

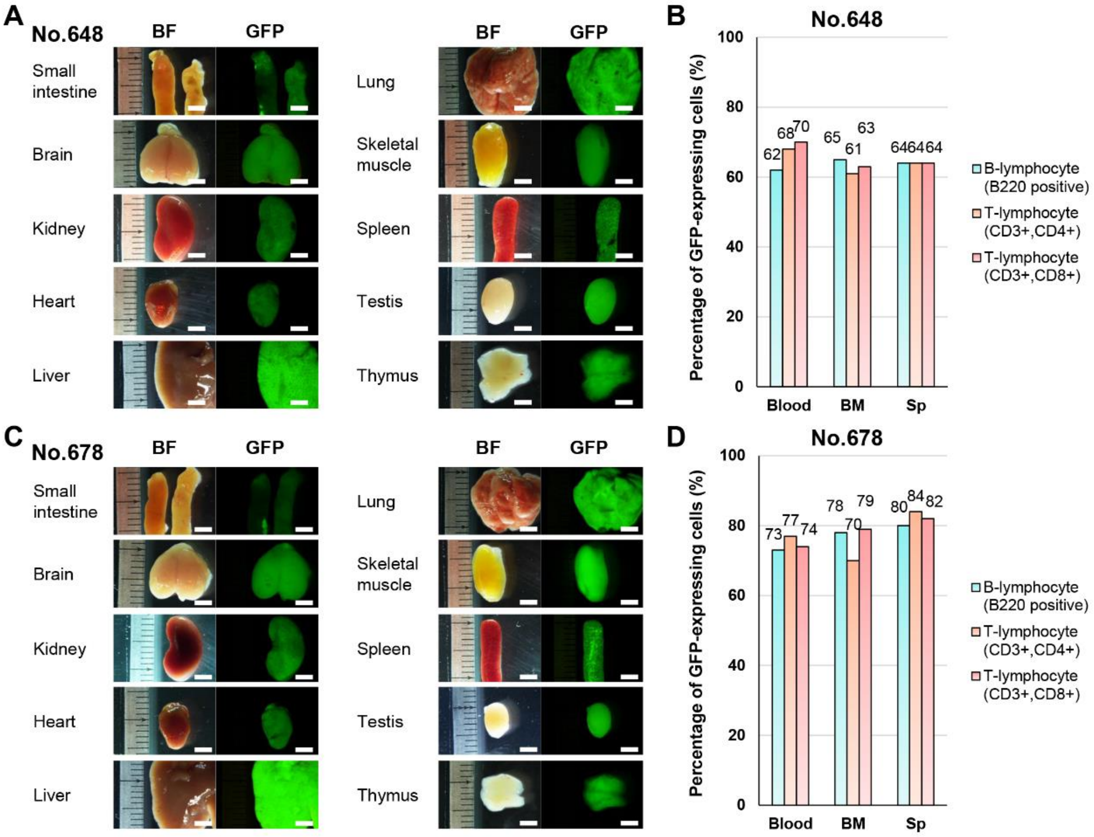

Supplementary Figure S4. Retention rate of 10MAC1 in Tc mice with low percentages of GFP-positive blood lymphocytes

(A) and (C) GFP images of different tissues from 10MAC1 Tc mice with low percentages of GFP-positive blood lymphocytes (64.72% and 74.63%, respectively). GFP expression indicates the presence of 10MAC1. Exposure times for each GFP image of small intestine, brain, kidney, heart, liver, lung, skeletal muscle, spleen, testis, and thymus were 100, 500, 200, 100, 400, 700, 100, 400, 200 and 200 ms, respectively. Scale bar: 3 mm. (B) and (D) Percentages of GFP-expressing lymphocyte subsets from blood, bone marrow and spleen from 10MAC1 Tc mice with low percentages of GFP-positive cells. Lower retention rate of the 10MAC1 in the peripheral blood reflected the lower retention rates among different tissues.

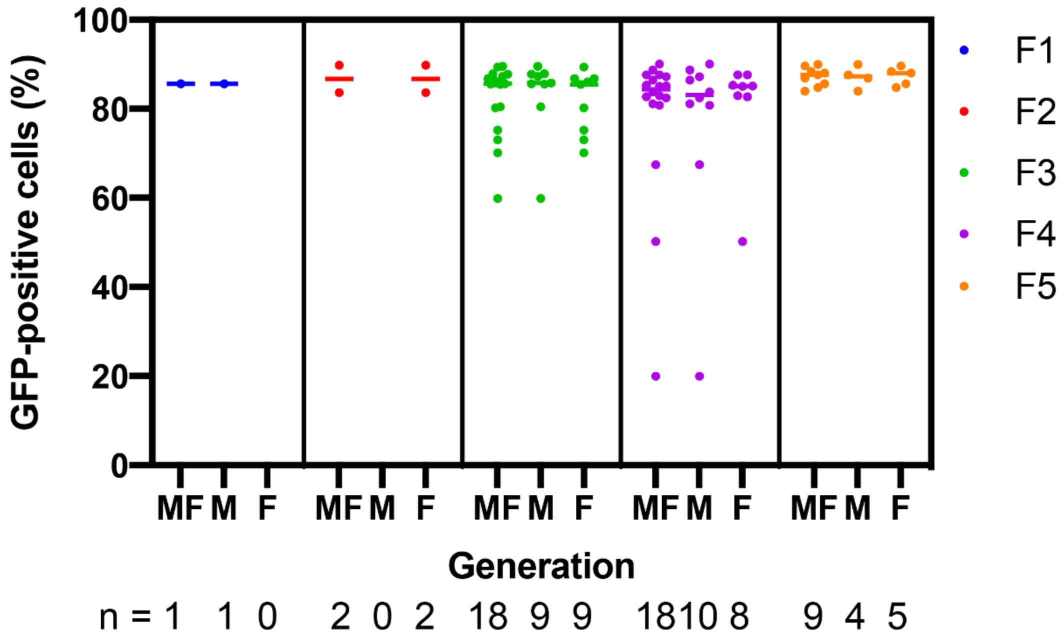

Supplementary Figure S5. Retention rates of 10MAC1 in lymphocytes of Tc mice through generations

Retention rates of 10MAC1 in lymphocytes from peripheral blood of Tc mouse strain were determined as percentages by FCM analysis monitoring GFP-positive cells through generations (F1-5). M: male. F: Female.

Supplementary Table S1. Primer sequences for genomic PCR analyses

Gene, vector or aim	Primer name (forward)	Forward primer (5'-3')	Primer name (reverse)	Reverse primer (5'-3')	Product size
pBS-TEL/puro_10MAC	AscI_m10T F2	TCGAGGCGCGCCAGCCTTCTAGGGAACAGGAGATGTTCAA	BamHI_m10T R3	TCGAGGATCCGCCTTGAGTGGGGTTCTAGTCATCTTTC	5.6 kb
Telomere truncation	m10 F6	AACTACCCAGTTCTGCATTTGGTGTGAG	Puro I	GAGCTGCAAGAACTCTTCCTCACG	7.0 kb
Gm8155	Gm8155 F	ACCCCTCGAACCCCTATTGC	Gm8155 R	CACGCCATCGGTGATGGATA	192 bp
lyd	lyd F	TGGGATGACCCCCACTTCTTT	lyd R	TTTTGGCCTCTTGCCCCATA	192 bp
Plekhg1	Plekhg1 F	TGGATGGGTTTCAATGCCACT	Plekhg1 R	GGCATTCTCCCCTGTTGTGG	159 bp
p10MAC1, 10MAC1 construction	KpnI_m10 LA F	TCGAGGTACCTCTAAGTCAGGGAAAGATCCCCTTCTTG	XhoI_m10 LA R	TCGACTCGAGGACCATGAAGATGGTCCAACTAAAGCAA	2.0 kb
p10MAC1	SalI_m10 RA F	TCGAGTCGACCACTGCTCTTTCTTTAGTTACATGCAGCCC	NotI_m10 RA R	TCGAGCGGCCGCATTCTTGCCAAGCTACTCTTCCGAGCTA	4.4 kb
10MAC1 construction	m10 F1	TGAGAAATACCGAATGGCAGAGAAACAC	EGFP-F	CCTGAAGTTCATCTGCACCA	5.0 kb
10MAC1 construction	kj neo	CATCGCCTTCTATCGCCTTCTTGACG	m10 R2	GAGAGGAGGGAAGCTTGATGAGAAAATG	7.0 kb
p10MAC2, p10MAC3	NotI_m10 LA F	TCGAGCGGCCGCTCTAAGTCAGGGAAAGATCCCCTTCTTG	SalI_m10 LA R	TCGAGTCGACGACCATGAAGATGGTCCAACTAAAGCAA	2.0 kb
p10MAC2, p10MAC3	ClaI_m10 RA F	TCGAATCGATCACTGCTCTTTCTTTAGTTACATGCAGCCC	ClaI_m10 RA R	TCGAATCGATATTCTTGCCAAGCTACTCTTCCGAGCTA	4.4 kb
10MAC2 construction	m10 F1	TGAGAAATACCGAATGGCAGAGAAACAC	10MAC R1	CTCTTCAGCAATATCACGGGTAGCCAAC	4.6 kb
10MAC2 construction	10MAC F1	TGCTTGCATTGTATGTCTGGCTATTCTG	m10 R2	GAGAGGAGGGAAGCTTGATGAGAAAATG	4.9 kb
10MAC3 construction	m10 F1	TGAGAAATACCGAATGGCAGAGAAACAC	EGFP-R	TGCTCAGGTAGTGGTTGTCG	7.0 kb
10MAC3 construction	10MAC F1	TGCTTGCATTGTATGTCTGGCTATTCTG	m10 R2	GAGAGGAGGGAAGCTTGATGAGAAAATG	4.9 kb
CENPH	CENPH Fw	TTCCCCATGAAGTGCAGCAA	CENPH Rv	AAACCACCGTGCAGTGCAGA	250 bp
Furin	Furin-F	ACTCAGAGATCCACTGCACCAGGATCCAAGGGAGG	Furin-R	CCGCTCGAGCGGCTACACCACAGACACCATTGTTGGCTACTGCTGCC	272 bp
Cre/loxP system	TRANS L1	TGGAGGCCATAAACAAGAAGAC	TRANS R1	CCCCTTGACCCAGAAATTCCA	408 bp

Supplementary Table S2. Confirmation of gene loading by PCR

	TRANS L1/R1	X3.1IEGFPI AnJ F1/AnJ_lox161R	FISH analysis
CHO 10MAC1 X6.1	24/24 (100%)	N.D.	N.D.
CHO 10MAC2 X3.1-I-EGFP-I	23/23 (100%)	18/21 (85.7%)	3/6 (50%)
CHO 10MAC3 X3.1-I-tdTomato-I	24/24 (100%)	23/24 (95.8%)	1/5 (20%)
